# Robotic lateral cervical lymph node dissection via bilateral axillo-breast approach for papillary thyroid carcinoma: a single-center experience of 260 cases

**DOI:** 10.1007/s11701-019-00986-3

**Published:** 2019-06-20

**Authors:** Qingqing He, Jian Zhu, Dayong Zhuang, Ziyi Fan, Luming Zheng, Peng Zhou, Fang Yu, Gang Wang, Gaofeng Ni, Xuefeng Dong, Meng Wang, Xiaolei Li, Changrui Liu, Dan Wang, Tao Yue, Lei Hou, Mengdi Wang, Dandan Li

**Affiliations:** Department of Thyroid and Breast Surgery, 960th Hospital of the People’s Liberation Army, No. 25 Shifan Road, Jinan, 250031 People’s Republic of China

**Keywords:** Papillary thyroid carcinoma, Robotic thyroidectomy, Bilateral axillo-breast approach, Robotic lateral cervical lymph node dissection

## Abstract

To evaluate the feasibility and safety of robotic lateral cervical lymph node dissection via BABA, 260 thyroid cancer patients with suspected level II, III, IV, and V_b_ lymph node metastasis were selected. The lateral cervical compartment was exposed by splitting the sternocleidomastoid muscle longitudinally, and separating between the strap muscles and the anterior margin of the sternocleidomastoid muscle. The procedure was completed in 260 patients. Mean time for robotic lateral node dissection took 80 ± 21 min. The wound catheter was removed 6.3 days. Postoperative transient symptomatic hypocalcemia was observed in 51 patients, transient hoarseness in three, seroma in three, chyle leakage in two, and tracheal injury in one. 124 patients were confirmed to have lymph node metastasis on final pathological report. Average postoperative hospital stay was 6.5 days. Robotic lateral neck dissection by BABA is the acceptable operative alternative for thyroid cancer patients who wished to keep their surgical history private.

## Introduction

It was George W. Crile who was the first to describe radical neck dissection in 1906. Recent innovation in the surgical technique of thyroidectomy has offered the opportunity for the patients to stay away from these prominent neck incision scars. Especially, with the advent of robotic surgical system (Intuitive Surgical, Sunnyvale, CA), many have adopted the concept of remote-access surgery and developed various robotic thyroidectomy techniques [1–3].

In China, bilateral axillo-breast approach (BABA) was utilized in most cases of robotic total thyroidectomy. BABA robotic thyroidectomy makes four tiny incisions (5–12 mm) at areolae and skin creases of the axillae, and can perform bilateral central/lateral cervical lymph node dissection (levels II, III, IV, and V_b_) effectively, without sacrificing surgical safety parameters [4–7]. However, robotic lateral cervical lymph node dissection remains at an early stage, and further evaluations are required.

## Methods

### Patient eligibility and study design

Since January 2014, we have completed 800 cases of robotic thyroidectomy. Here, we present our experience of 260 patients who received robotic total thyroidectomy with lateral cervical lymph node dissection via BABA between Jun 2014 and May 2018. General clinical information of the patients is outlined in Table 1.

**Table 1:** Clinical characteristics and surgical outcomes of the patients

Items	RLCLND (*n* = 260)
Mean age (years)	41.2 ± 11.9
Male/female, (*n*)	63:197
BMI (kg/m^2^)	23.9 ± 3.7
Primary tumor size (mm)	10.7 ± 6.9
Multicentricity/multifocality (*n*)	97
Type of surgery	
Total thyroidectomy with lateral RLCLND (*n*)	239
Total thyroidectomy with bilateral RLCLND (*n*)	21
Conversion to open surgery	0
Total number of removed lateral nodes (*n*)	17.9 ± 8.6
Lateral node metastasis (*n*)	124
Total operating time (min)	201 ± 63
Postoperative hospital stay (d)	6.5 ± 2.6
Mean drain days (d)	6.3 ± 1.1
Postoperative complications	
Postoperative bleeding (*n*)	0
Subcutaneous emphysema	0
Pneumomediastinum	0
Hypoparathyroidism	
Temporary (*n*)	51
Permanent (*n*)	0
RLN paralysis	
Temporary (*n*)	3
Permanent (*n*)	0
Seroma	3
Skin burn	0
Flap necrosis	0
Hematoma	0
Tracheal fistula	1
Wound infection	1
Chyle leakage (*n*)	2
Postoperative Tg level (ng/mL)	0.81 ± 0.2
Cosmetic satisfaction score	4.68 ± 0.35
Mean follow-up (months)	28.6 ± 8.3

*BMI* body mass index, *RLN* recurrent laryngeal nerve, *Tg* thyroglobulin, *RLCLND* robotic lateral cervical lymph node dissection

The inclusion criteria for robotic total thyroidectomy with lateral cervical lymph node dissection via BABA were as follows: (1) patients of papillary thyroid cancer with suspicious or cytologically confirmed lateral neck metastasis (by FNAC), central lymph node metastasis (diagnosed by frozen section examination during the operation), or evident lateral neck lymph node metastasis on preoperative imaging studies and (2) patients with no previous history of treatment for neck surgery of any kind, whereas the exclusion criteria were: (1) metastatic lymph nodes were fused with each other or fixed in the neck, or (2) patients with thyroid carcinomas that showed gross invasion to local structures or extensive extrathyroidal capsular spread (such as trachea, esophagus, and recurrent laryngeal nerve), or (3) patients with the metastatic lymph nodes encase the common carotid artery or internal jugular vein or liquefied lymph nodes, or (4) the suspicious lymph nodes are located beneath the clavicle. To assess the extent of disease, neck ultrasonography with fine-needle aspiration, neck CT, MRI, or PET-CT was performed as preoperative evaluation. All patients were given full information of the possible treatment options for their thyroid cancer, including the advantages and disadvantages of conventional open transcervical approach and robotic surgery via BABA, and provided written, informed consents before the surgery.

The day before robotic surgery, 0.1–0.2 ml of carbon nanoparticles’ suspension injection (Chongqing Lummy Pharmaceuticals, China) was injected into the thyroid gland under the guidance of ultrasound, in the purpose of leaving the parathyroid glands unstained, while staining the lymph nodes of the center compartment and lateral cervical [5].

### Surgical procedures

#### Position

**Fig. 1:**
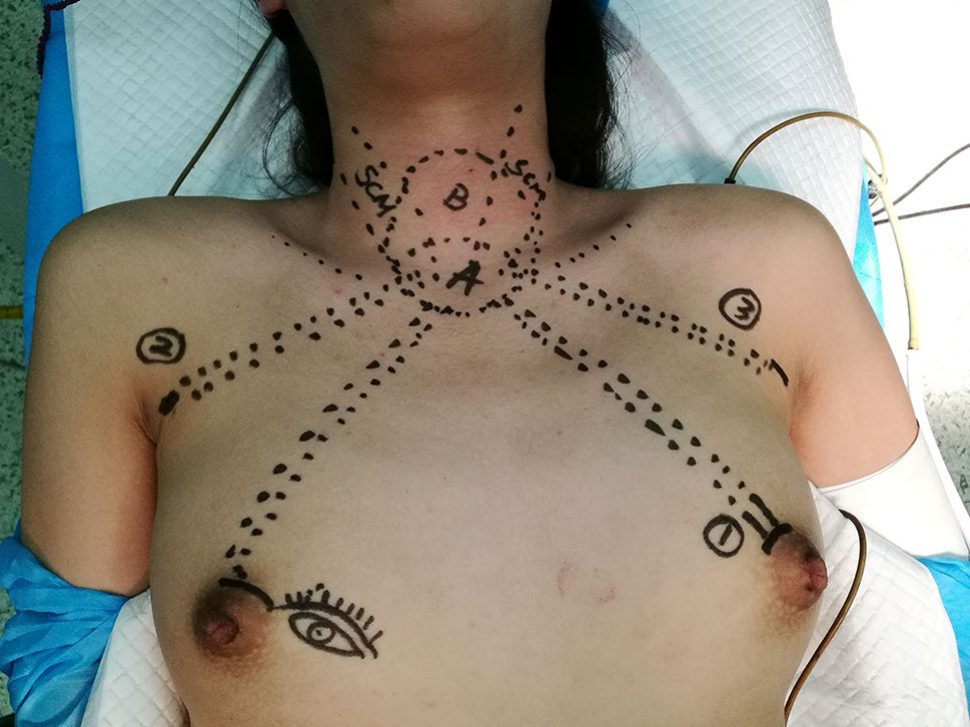
Drawing the instrument arm trajectory lines

Under general endotracheal anesthesia, patient was placed on the operating table in the supine position with the neck slightly extended and both arms slightly abducted to allow insertion of the axillary port. After draping, we draw the instrument arm trajectory lines and working area on patient’s chest and neck for reference (Fig. 1).

#### Flap design and dissection

**Fig. 2:**
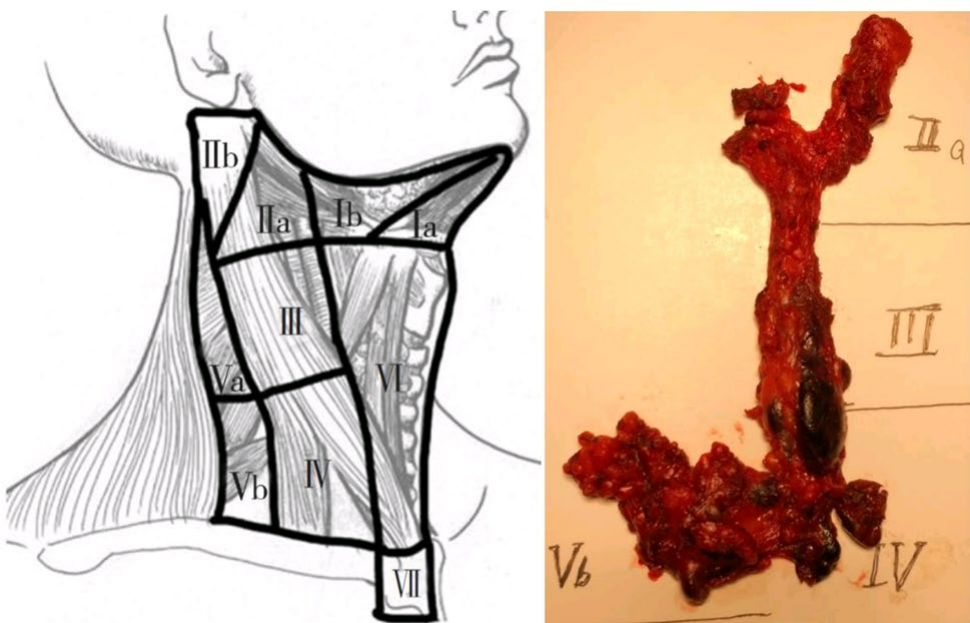
Surgical landmarks of lateral neck node

Draw guidelines along the landmarks of the chest and the neck, such as the linea alba, trachea, thyroid cartilage, cricoid cartilage, anterior border of the sternocleidomastoid (SCM) muscle, the clavicles (Fig. 2), suprasternal notch, circumareolar and axillary incisions, and trajectory lines from the incision to cricoid cartilage as described in the previous chapter [2, 5].

Usually, 5 mm Maryland dissectors are docked in the left axillary port, and the Prograsp forceps are docked in the right axillary port. Maintain low pressure (5–6 mmHg) with high flow of carbon dioxide (15 l/min) to create surgical field. The working space for BABA robotic lateral neck dissection should be made wider than the flap for usual robotic thyroidectomy. It should be extended over the lateral border of the ipsilateral SCM muscle laterally and to the lower border of the submandibular gland and posterior belly of digastric muscle superiorly. It is important that sufficient working space can be created for comfortable movements of the robotic arms through the BABA during both total thyroidectomy and neck dissection. During flap dissection to the lateral side, identifying and protecting the great auricular nerve are also of vital importance.

**Fig. 3:**
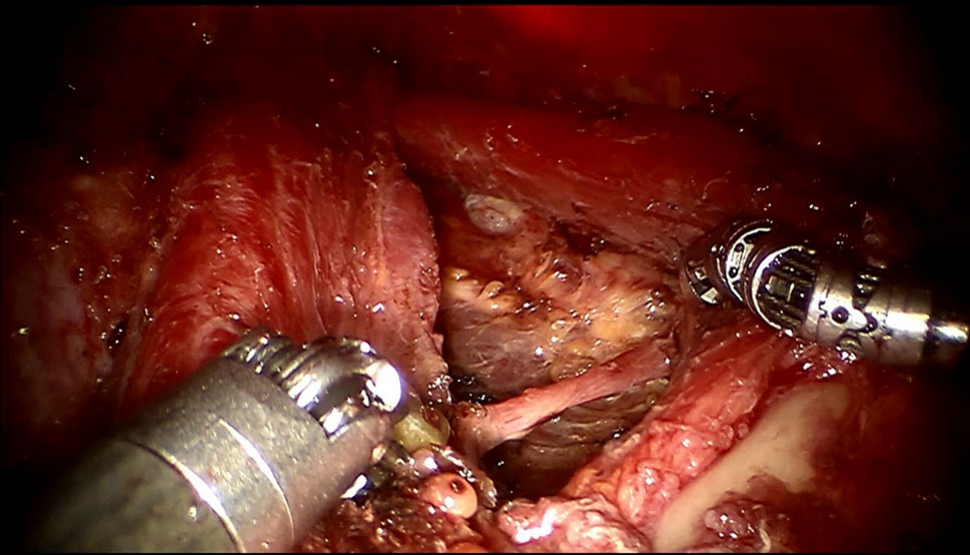
Omohyoid muscle and the clavicles were revealed

After completing total thyroidectomy and central compartment dissection, dissection of ipsilateral or bilateral levels II, III, IV, and V_b_ was performed (Fig. 3). The steps of robotic lateral cervical node dissection were similar to those of conventional surgery. After the working space was created, the lateral cervical compartment was exposed by splitting the SCM longitudinally and separation between the strap muscles and the anterior margin of the SCM is needed. The entire medial border of the SCM muscle is dissected with lateral traction of the Maryland forceps. The omohyoid muscle is identified and protected, and its inferior belly is dissociated by a Harmonic curved shear. The omohyoid muscle is lifted upward and the internal jugular vein is revealed. The omohyoid muscle is retained during the operation to maintain the appearance of the lateral area (Fig. 2). After the medial border of SCM is fully exposed, the muscle is pulled laterally using small gauze roll by 5 mm Maryland dissector.

#### Lateral cervical lymph node dissection

The lateral cervical compartment was exposed by splitting SCM longitudinally, and separating between the strap muscles and the anterior margin of SCM. One should notice that the harmonic scalpel has a limited range of motion and lacks side-bending capabilities; at the same time, the camera is not flexible and the surgical view is limited. For better visualization the lowermost sub-clavicular area, the robotic 30° down camera is set as possible as high. A slight clockwise or counterclockwise rotation of the camera port is also recommended. The internal jugular vein is drawn medially using the Prograsp forceps. The soft tissue with lymph nodes is detached from the anterior surface of the internal jugular vein to the posterior aspect of the internal jugular vein until the common carotid artery and vagus nerve are identified. Inferior dissection of level IV is performed with preservation of the transverse cervical artery and the phrenic nerve. Especially, in the left level IV dissection, branches of the thoracic duct are identified by the 15 times magnified view of the da Vinci Si system. Thoracic duct needs to be preserved. Main trunk of the thoracic duct can be ligated using absorbable sutures or robotic Hem-o-lok clips to prevent postoperative chyle leakage. Small branches of lymphatic ducts can be managed using harmonic curved shears. Careful dissection of level IV is required to preserve the phrenic nerve. Do not injure the phrenic nerve which is always deep to the transverse cervical artery, do not enter the deep space beneath the transverse cervical artery unless absolutely necessary.

The dissection is extended to level III after dissecting level IV and V_b_. The vagus nerve and the phrenic nerve that are identified at the level IV dissection should be traced carefully not to pose any injury. Care should be taken to preserve C2, C3, and C4 as far as possible (Fig. 3). Dissection of the level III compartment is performed carefully not to perforate the internal jugular vein.

IIa and III compartments were exposed by separating between the strap muscles and the anterior margin of SCM. The dissection proceeds until the posterior belly of the digastric muscle is exposed superiorly. The spinal accessary nerve runs under the internal jugular vein mostly, but can be found over the internal jugular vein sometime, and should be preserved at this point. The spinal accessary nerve can be confirmed with the current of 3.0–5.0 mA of neuromonitor (Shanghai NCC Medical Co., LTD, China).

#### Closure

The resected specimen was extracted through enlarging the axillary skin incision using a specimen pouch. Meticulous hemostasis is needed. The surgical area was irrigated by 3500 ml sterile distilled water (42 °C) to reduce tissue debris to prevent tumor tissue planting. Suture the linea alba is needed in most cases. One vacuum-assisted draining system is inserted in levels IV area though ipsilateral axilla tunnel, and the other one should be placed in the thyroid bed though the areolar tunnel. The skin incision was closed with 5–0 absorbable sutures using an atraumatic needle, and wounds are covered by placement of Steri–Strips. The drains are removed when the patient is discharged, usually on postoperative days 4–7.

### Statistical analysis and followed up

For descriptive statistics of quantitative variables, mean ± standard deviation and range were used to describe central tendency and dispersion. For analysis of the differences in proportions was used. Follow up by outpatient, phone, email, and WeChat. Tumor recurrence is regularly monitored by ultrasonography (ultrasound and ultrasound-guided fine-needle aspiration citology), measuring Tg or FNA-Tg concentration at the outpatient clinic. Neither iatrogenic implantation nor metastasis occurred in drain tract/chest wall was found by inspection or ultrasound during follow-up.

Cosmetic satisfaction at 3 months postoperatively was assessed using a five-point scale (extremely satisfied, satisfied, acceptable, dissatisfied, or extremely dissatisfied).

## Results

**Fig. 4:**
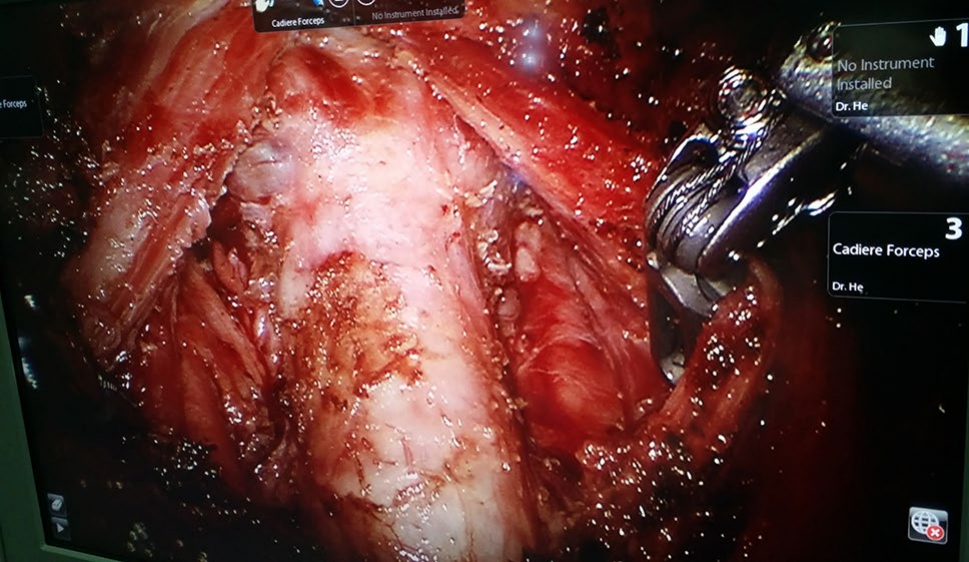
Ultrasonic scalpel burning tracheal wall

**Fig. 5:**
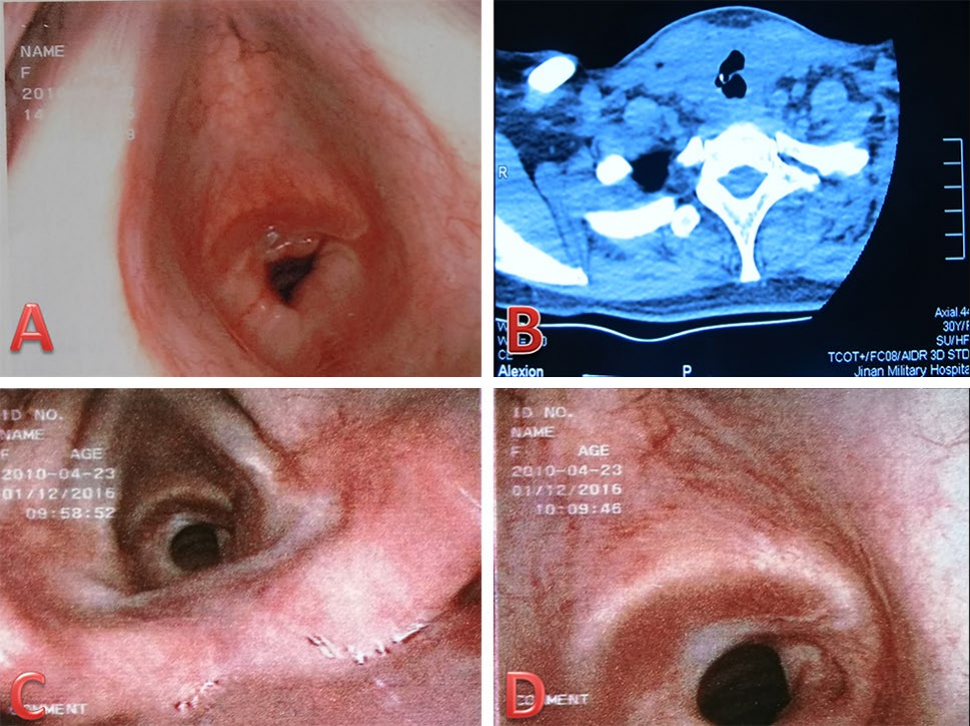
Tracheal fistula and tracheal annular stenosis

All the patients received thyroid hormone replacement therapy for life-long time. All complications were recorded, including one patient who had severe scar constitution underwent tracheal fistula 5 days after surgery (Fig. 4). During the operation, thyroid cancer was found to infiltrate the trachea, and ultrasonic knife was used to cauterize the infiltrated tracheal wall (Fig. 4), which was managed conservatively without the need for further surgical intervention (Fig. 5). All cases of transient hypocalcemia resolved with the need for calcium or vitamin D supplementation in 2 months. Chyle leakages were managed conservatively with a fat-free diet and by placing a compressive dressing over the supraclavicular fossa without the need for further surgical intervention, symptom resolved within 2 weeks. There are three patients with RLN paralysis, and this was determined on laryngoscopy.

Lateral cervical lymph node dissection (unilateral or bilateral levels II, III, IV, and V_b_) via BABA was completed successfully in 260 patients without any significant intraoperative complications or conversion to open/other approaches (Table 1). Mean operative time was 201 ± 63 min, in which mean console time for robotic lateral neck dissection took 80 ± 21 min. The wound catheter was removed 6.3 ± 1.1 days after surgery. This series was comprised of patients [(body mass index, BMI): 20–37.7 kg/m^2^, average 23.9 ± 3.7 kg/m^2^] with thyroid nodules (average 12 mm) across all age groups (41.2 ± 11.9 years, range 15–68 years). Majority of patients (*n* = 146,56.2%) had papillary thyroid microcarcinoma on final surgical pathology, rest 43.8% had papillary thyroid cancer. In this study, postoperative transient symptomatic hypocalcemia was observed in 51 patients (19.6%), postoperative transient hoarseness in 3 (1.15%), seroma in 3 (1.15%), chyle leakage in 2 (0.7%), and tracheal fistula (caused by ultrasonic knife cauterization) in 1 (0.4%) (Figs. 4, 5). There was no permanent hypocalcemia and hoarseness, no pneumothorax or carotid artery injury, and no traction injury. No bleeding or hematoma was encountered. No implantation or metastasis occurred in drain tract or chest wall. 124 patients (47.7%) had lymph node metastasis in the lateral compartment confirmed on the final pathological report. Mean lymph node yield in the lateral compartment was 17.9 (17.9 ± 8.6, range 7–41). Average postoperative hospital stay was 6.5 ± 2.6 days (range 3–11 days).

All patients are being followed up (average 28.6 ± 8.3 months, range 1–48 months) on a regular basis. All cases experienced sensory impairment in the neck, chest, or the nipples after BABA procedures, but such touch and pain disturbance could be normalized completely by 4–12 months. Cosmetic satisfaction score was 4.68 ± 0.35. No patient who had issues with postoperative paresthesias, pain, breast asymmetry, the brachial plexus injuries, or neck stiffness.

In one 27-year-old male patient’s 13 months postoperatively ultrasound examination, swollen lymph nodes on the left side of the level IV were revealed with serum Tg less than 0.2 ng/mL. The combination of FNAC and FNA-Tg (eluent Tg > 300 ng/mL) diagnosed lymph node metastasis and the patient received open surgery later to remove one metastasis lymph node. 118 patients have received high-dose (104–150 mCi) radioiodine ablation therapy after the operation.

## Discussion

The most commonly used approaches are BABA in China [5–7], the symmetry of the BABA system enables bilateral central/lateral neck dissection. Other new approaches to robotic thyroidectomy also have been described, including axillary breast, retroauricular, and transoral [8–12]. The BABA is a multidirectional approach in which the midline is the viewpoint, whereas the other ones are lateral approaches. Majority of patients had microcarcinoma, yet all had lateral neck node dissection, with overall only 47.7% small positive nodes in the specimen. With such low yield, most of the neck dissections were done prophylactically. However, most microcarcinomas would only require lobectomy and rarely need prophylactic central neck node dissection and none would need prophylactic lateral neck dissection [13, 14]. It is recommend in China to perform prophylactic central neck node dissection, which is discourage by 2015 ATA guidelines. Prophylactic lateral neck node dissection is specifically discouraged by the ATA guidelines [13]. In 2016, the American Thyroid Association again published a statement, indicating that remote-access thyroidectomy may be performed safely in high-volume centers [15], and there acknowledged the role of robotic thyroidectomy in selected patients and emphasized the importance of strict selection criteria [14–16]. The selection of approach is largely dependent on the training, skill, and preference of the surgeon and/or patient [17].

Compared to the conventional open approach, robotic total thyroidectomy with lateral cervical lymph node dissection only requires one 1.2 cm and one 0.8 cm incisions in bilateral areolas, one 0.8 cm and one 0.5 cm incisions at the axillary crease. These wounds could be well-hidden with everyday clothing, which was especially important in patients who wished to keep their surgical history private. The essence of robotic thyroidectomy by BABA entails better patients’ satisfaction on cosmetic outcomes. Appropriate positioning of the patient and configuration of the robotic instruments are keys to successful operation. It is important that sufficient working space be created for comfortable movements of the robotic arms through the axillary–areolar ports during both total thyroidectomy and neck dissection. In comparison with traditional transcervical open methods, by placing the surgical incision in the axillary and circumareolar, an obvious cervical incision would be completely refrained. Robotic thyroidectomy system combines the unique benefits of the surgical robot and remote-access thyroidectomy provides the welfares of excellent 3-D visualization,stable and magnified operative view, and improved surgical dexterity. It has been proved to have comparable surgical safety and oncological completeness with traditional open operation, and creates smaller injury (psychological and physical injury), better functional preservation (including neck function, parathyroid glands, and laryngeal nerve), and significantly cosmetic satisfaction. Indeed, many patients are highly concerned on the scar associated with thyroid surgery and with the difficulty hiding this scar. At present, the amount of Chinese community medical institutions and family care physicians cannot meet the need of individual patients’ postoperative follow-up treatment, and most thyroid cancer patients stay in hospital after surgery until the removal of the neck drainage pipe discharge. Therefore, postoperative hospital stay usually was 6 days. Late follow-up results indicated that splitting the SCM longitudinally during neck dissection did not increase postoperative pain and morbidity, such as neck stiffness (especially with bilateral procedure).

Using convenient robotic instruments helped to overcome some of the limitations of the endoscopic procedures such as reduced range of motion, long and rigid instruments, chopsticks effect, and impaired eye–hand coordination (while relying on an unstable 2D view). Robotic thyroidectomy has become increasingly popular around the world attracting both surgeons and patients searching for new and innovative procedures and allowing for the removal of thyroid with an excellent cosmetic result when compared to the open thyroidectomy. Many reports have described the safety of the remote-access robotic thyroidectomy procedures and have demonstrated comparable oncologic outcomes between the robotic and open thyroidectomy [12, 16].

From surgeon’s point of view, the bilateral axillo-breast approach would be more comfortable due to familiar local anatomical structures, easier to address the thyroid gland, trachea, recurrent laryngeal nerve, and the spinal accessory nerve, and lymph node tissues at the central or bilateral cervical lymph node could be easily reached due to the decreased area of dissection compared with the transaxillary, transoral, or retroauricular approach [16]. BABA most closely resembles traditional thyroid surgery, as it provides a midline access to the symmetrical view of both lobes. Adopting carbon nanoparticles in negative development of the parathyroid gland helps to identify and protect the parathyroid glands and remove more lymph nodes and shorten the operation time. This operative procedure, however, has disadvantages of longer operation times, use of CO_2_ insufflation, no force and haptic feedback, and the higher costs. It is contemplated that the operation time would be significantly decreased through accumulation of surgical experience. The insufflation of high volume of carbon dioxide (15 l/min) to maintain low pressure (5–6 mmHg) can obtain satisfactory surgical field, and can avoid the occurrence of subcutaneous emphysema. Tracheal injury rate was reported in one patient. Tracheal fistula is an uncommon complication. It is something to be concern about with this approach. The reason for consideration is that in the case of patients with severe scar constitution and lack of haptic feedback, severe burning of the trachea by ultrasonic knife leads to tracheal wall necrosis and tracheal fistula. However, most experienced surgeons may compensate for this drawback using visual thinking. Despite all progress, the financial commitment and time sacrifice are the major limitations for a broad implementation of robotic thyroidectomy into daily routine. Despite our promising operative results, an expanded population from careful selection of eligible patients is mandatory, and future prospective trials should be conducted to evaluate long-term outcomes and to overcome potential limitations [17–24].

Traditional open thyroidectomy continues to be the most safe and effective, standard operation for thyroid during past century. However, it leaves a noticeable scar in a highly visible area which is undesirable for many patients. Since the late twentieth century, people have paid great attention to the quality of life. Neck scar is a life-long stigma for some patients. The advent of cosmesis intended thyroid surgery, and no scar to the neck procedures helps get rid of cancer safely while keeping patients in beauty. All cases experienced minimal numbness and tingling in the neck and chest, but such sensory disturbance could be normalized completely by 4–12 months. Through technical reinforcement along with experience accumulation and development of robotic instruments, robotic thyroidectomy can overcome the possible limitation of safety and oncological radicalness and can also be a new treatment modality for papillary thyroid cancer. In addition, guidelines are needed to reduce trial errors [17].

## Conclusions

Robotic thyroidectomy is not for every patient, surgeon, or hospital. Considered and measured patient selection is paramount. Robotic thyroidectomy with lateral cervical lymph node dissection via BABA may be an appropriate option for the carefully selected patients who are committed to completely avoiding a neck scar.
